# A rift between implicit and explicit conditioned valence in human pain relief learning

**DOI:** 10.1098/rspb.2010.0103

**Published:** 2010-03-31

**Authors:** Marta Andreatta, Andreas Mühlberger, Ayse Yarali, Bertram Gerber, Paul Pauli

**Affiliations:** 1 Department of Psychology, University of Würzburg, Würzburg, Germany; 2 Department of Neurobiology and Genetics, University of Würzburg, Würzburg, Germany

**Keywords:** relief, pain, startle reflex

## Abstract

Pain is aversive, but does the cessation of pain (‘relief’) have a reward-like effect? Indeed, fruitflies avoid an odour previously presented before a painful event, but approach an odour previously presented after a painful event. Thus, event-timing may turn punishment to reward. However, is event-timing also crucial in humans who can have explicit cognitions about associations? Here, we show that stimuli associated with pain-relief acquire positive implicit valence but are explicitly rated as aversive. Specifically, the startle response, an evolutionarily conserved defence reflex, is attenuated by stimuli that had previously followed a painful event, indicating implicit positive valence of the conditioned stimulus; nevertheless, participants explicitly evaluate these stimuli as ‘emotionally negative’. These results demonstrate a rift between the implicit and explicit conditioned valence induced by pain relief. They might explain why humans in some cases are attracted by conditioned stimuli despite explicitly judging them as negative.

## 1. Introduction

Primary reinforcers are either positive (e.g. food) or negative (e.g. noxious events), and stimuli associated with primary reinforcers will generally acquire similar qualities.

However, event-timing is a crucial determinant for this process. For example, Dickinson & Dearing (1979) pointed out that a stimulus predicting the absence of a threat can have similar behavioural effects as a stimulus predicting an appetitive event. Corroborating this assumption, fruitflies were found to avoid an odour previously associated with the onset of an electric shock, as it predicts danger, but approach an odour previously associated with the shock's offset, as it predicts safety (Tanimoto *et al*. 2004; Yarali *et al*. 2008). Using such a backward conditioning paradigm, similar results have been found in mice and rats as well (Cunningham *et al*. 2002; Salvy *et al*. 2004). In humans, fear studies corroborate that a stimulus preceding a shock becomes a predictor for danger (Lipp *et al*. 1994; Grillon 2002). However, it remains unknown whether in humans a stimulus following an aversive event later on predicts safety.

Notably, dual-process theories (Strack & Deutsch 2004; Bechara 2005) propose that human behaviour is determined by the output of two systems, an impulsive, implicit system working on associative principles, and a system operating on the basis of reflective, explicit knowledge about facts and values. Importantly, these systems can operate in a synergistic or antagonistic fashion and it remains to be clarified how event-timing affects these systems and/or their interaction. Understanding event-timing in humans and its potentially dissociated effects on the impulsive and reflective system may be relevant for psychopathologies like drug addiction (Koob & LeMoal 2001; Weiss 2005), or anxiety disorders (Bouton *et al*. 2001; Mineka & Oehlberg 2008): both disorders are characterized by emotionally intensely negative episodes such as drug withdrawal and anxiety attacks, respectively, and stimuli associated with the offset of these events may become appetitive.

To examine the effects of event-timing in humans, we compared three groups of participants who had undergone stimulus-discrimination learning reinforced by an aversive unconditioned event, i.e. a moderately painful electric shock; what differed between the three experimental groups is the relative timing of stimulus and this shock. On the one hand, the valence of the stimulus associated with this electric shock is assessed on the basis of explicit, subjective reports. On the other hand, the implicit valence of the stimulus is assessed by probing for its capacity to modulate the startle response (Lang *et al*. 1998); this is an especially suitable method because it allows us to assess both positive and negative valence effects within one setting, and because it allows translational studies between humans and rodents. That is, the startle response is an evolutionarily conserved defence reflex, the neural mechanisms of which have been studied in detail (Koch 1999; Davis 2006). Significantly, if animals or humans are trained to associate a stimulus (e.g. a light) with a painful electric shock, the startle response amplitude is potentiated in the presence of that shock-predicting stimulus (Lipp *et al*. 1994; Koch 1999; Grillon 2002; Davis 2006). Such an associative increase of startle amplitude indicates fear and is mediated by connections from the amygdala impinging upon the startle-reflex circuitry (Davis 2006). However, when the stimulus is trained to predict a rewarding sucrose solution, the startle amplitude is attenuated in its presence (Koch *et al*. 1996; Schneider & Spanagel 2008). This associative attenuation may involve dopaminergic projections of the nucleus accumbens (NAcc) onto the startle circuitry (Schultz 2006). Also in humans, attenuation of the startle response has been found in the presence of pleasant stimuli (Lang *et al*. 1998) or stimuli signalling monetary gain (Skolnick & Davidson 2002). Therefore, we reasoned that a safety-predicting stimulus may attenuate the startle response in humans. Thus, regarding danger- or safety-predicting stimuli we complement explicit valence judgments with startle modulation as an implicit measure of valence.

## 2. Material and methods

### (a) Participants

A total of 101 healthy volunteers (68 females: mean age = 23.2 years, s.d. = 4.6, range = 18–43 years) were divided into three groups, which only differed in the relative timing of the conditioned stimulus (CS+) and the unconditioned stimulus (US) during *acquisition* (training phase): one group of 34 participants underwent forward delay conditioning (FORWARD: US onset 8 s after CS+ onset), one group of 34 participants a forward trace conditioning (CONTROL: US onset 14 s after CS+ onset), and the third group of 33 participants received backward conditioning (BACKWARD: US onset 6 s before CS+ onset). Participants were free of neurological, psychiatric or chronic pain diseases.

### (b) Stimulus material and apparatus

The US was a single unipolar electric shock of 200 ms duration generated by a battery-driven constant-current stimulator (maximum of 140 V and of 10 mA) delivered via a surface bar electrode (which consisted of two durable gold-plated stainless steel disc electrodes with 9 mm diameter and 30 mm spacing) attached to the left forearm (Neumann & Waters 2006). US intensity was assessed before the experimental session. Each participant received two series of electrical stimuli with ascending and two with descending intensity in steps of 0.5 mA (Reiff *et al*. 1999). Participants evaluated the intensity of each electrical stimulus on a rating scale ranging from 0 (no pain at all) until 10 (unbearable pain). The mean value of the intensities rated as ‘just noticeable pain’ (i.e. 4) was defined as pain threshold and increased by 1 mA.

CSs were three simple geometrical shapes (a square, a circle and a equilateral triangle), all solid yellow, 12 cm in width and 12 cm in height (see Lipp *et al*. 1994). These stimuli were always presented on a 19" black computer screen for 8 s. The screen was located 140 cm from the participants at eye level. Shapes could function either as a reinforced CS (CS+), which was always associated with the US (in either a FORWARD, CONTROL or BACKWARD way), as a non-reinforced CS (CS−), which was never associated with the US, or a new stimulus (NEW), which was presented only during the test phase (i.e. extinction). The shapes' functions were counterbalanced among participants.

The *startle stimulus* was a burst of white noise (50 ms, 105 dB) delivered binaurally with headphones. The eye-blink component of the startle response was measured through electromyography (EMG) of the left *orbicularis oculi* muscle with two 5 mm Ag/AgCl electrodes; one placed under the pupil of the left eye and the other approximately 1 cm lateral. Both the ground and the reference electrodes were placed on the forehead. Before attaching the electrodes, the skin was cleaned with alcohol and slightly abraded to keep all electrode impedances below 5 k*Ω* (measured with Vision Recorder V-Amp Edition Software). The raw signal was sampled at 400 Hz. Startle responses were registered continuously with a V-Amp 16 using Vision Recorder V-Amp Edition Software (v. 1.03.0004). EMG activity was filtered online with a 50 Hz notch filter to eliminate 50 Hz interference.

### (c) Procedure and experimental design

After having signed an informed consent form, participants were seated in a comfortable chair in a sound-attenuated room next to the experimenter room. After electrode attachment, the pain threshold was assessed. Participants were then informed that a series of geometrical shapes would be presented and that they should keep these pictures in their visual focus. Participants were also told that electrical stimuli would be delivered occasionally.

The experiment consisted of two phases, the training phase (i.e. acquisition) and the test phase (i.e. extinction). Additionally, before and after the training phase, the participants were asked to rate the three visual stimuli (CS+, CS− and NEW) in terms of their valence and arousal value. This *rating procedure* was realized as follows: first, the visual stimulus was presented for 1.2 s. Then, the valence and the arousal of the stimulus were assessed in succession. In both cases, a scale ranging from 1 to 9 appeared on the screen in front of the participants. For valence ratings, 1 was labelled ‘very unpleasant’ and 9 ‘very pleasant’; for arousal ratings, 1 was labelled ‘calm’ and 9 ‘exciting’. Participants had to respond by using a numeric keyboard. For analyses, valence and arousal data were transformed by subtracting 5 (as a consequence, negative values represent negative valence or low arousal, respectively, whereas positive values represent positive valence or high arousal; a value of zero represents ‘neutral’ ratings).

The *training phase* (acquisition) consisted of 32 trials: 16 presentations of CS+, always associated with the US and 16 presentations of CS−, never associated with US. The trial length varied between 28 and 44 s (mean of 36 s), the intertrial interval (ITI) varied between 20 and 30 s. The three experimental groups only differed in the time between CS+ onset and US onset (i.e. the interstimulus interval, ISI); for the FORWARD conditioning group, US onset coincided with CS+ offset, such that the US was delivered 8 s after CS+ onset (ISI = 8 s); for the BACKWARD conditioning group, US onset preceded CS+ onset by 6 s (ISI = −6 s); for the CONTROL conditioning group, US onset followed CS+ onset by 14 s (ISI = 14 s). No startle stimuli were delivered during the training phase.

The *test phase* (extinction) started with nine startle stimuli delivered every 7–15 s to decrease initial startle reactivity. Then, 48 extinction trials were run in a way identical to all participants; during the test phase, no US was delivered. Trial's length varied between 28 and 38 s (mean length 33 s), the ITI varied between 20 and 30 s. The three visual stimuli (CS+, CS− and NEW) were presented 16 times for 8 s each. In summary, 32 startle stimuli were delivered, eight in the presence of CS+, eight in the presence of CS− and eight in the presence of the NEW stimulus; thus, the respective visual stimuli were presented with or without the startle stimulus in half of the cases. These startle stimuli occurred 3–7 s after visual stimulus onset. In order to enhance the unpredictability of the startle stimuli, eight additional startle stimuli were delivered during the ITI (not analysed).

### (d) Data reduction and statistical analysis

Startle responses EMG data were analysed offline with the Brain Vision Analyser Software (v. 1.05, BrainProducts Inc.). Data were first filtered (low cut-off filter 28 Hz, high cut-off 500 Hz, moving average of 50 ms) and rectified. Then, startle response amplitude was determined for each trial as the peak startle response (the maximum in the 20–120 ms time window following the startle stimulus) relative to baseline defined as mean EMG activity over 50 ms preceding stimulus onset (see Grillon *et al*. 2006). Trials were excluded if the baseline EMG was not stable, or if the onset of the startle response was not within 20–60 ms after the startle probe onset. Startle response amplitude of each participant were standardized as a *z*-score (*z* = (*x* − *μ*)/*σ*), where *x* is a raw score, *μ* is the mean which is zero and *σ* is the standard deviation which is 1) in order to normalize data and to reduce the influence of between-subjects variability unrelated to psychological processes (see Blumenthal *et al*. 2005). Finally, mean startle response amplitude for each participant and each CS type (CS+, CS− and NEW) were calculated on the basis of this *z*-score.

Startle data were analysed with an analysis of variance (ANOVA) including the between-subjects factor group (FORWARD, BACKWARD, CONTROL) and the within-subjects factor stimulus (CS+, CS− and NEW); the ANOVA for the valence and arousal ratings had the additional within-factor time (BEFORE, AFTER acquisition). The *α* level was set at 0.05 for all statistical tests. Greenhouse–Geisser corrections (GG-ε) were used for main effects and interactions involving factors with more than two levels.

All data were analysed using SPSS for Windows (Release 17.0).

## 3. Results

**Figure 1.**
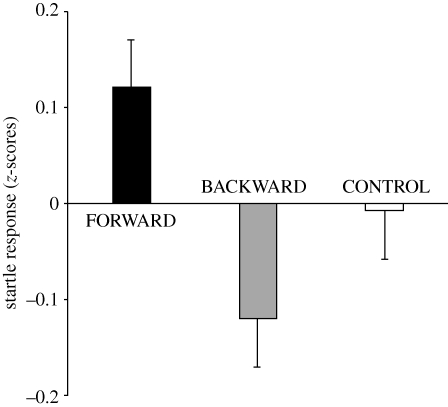
Bars represent mean (with standard errors) startle amplitudes in *z*-scores assessed in the test phase (i.e. extinction) in the presence of visual CS+; the black bar represents the FORWARD group, the grey bar the BACKWARD group, and the white bar the CONTROL group. Positive values indicate startle response potentiation; negative values startle attenuation relative to the mean. CS+ has been reinforced during the preceding training phase (i.e. the acquisition) with a different timing relative to the US (i.e. a mild electric shock). In the FORWARD and the BACKWARD group, the CS+ briefly preceded or followed the US, respectively, and consequently the CS+ had opposite effects on startle response modulation during test. In the CONTROL group, the CS+ preceded the US with a long delay and this did not subsequently affect the startle response.

Implicit valence ratings assayed by the modulation of startle revealed a significant interaction of stimulus × group (*F*_4,196_ = 3.49, *p* = 0.009; figure 1), underscoring the crucial role of the temporal sequence of events experienced during training; no other ANOVA effect reached significance. Follow-up tests (comparison of the group's mean with the overall mean of 0, i.e. the mean of the *z*-normalized distribution) indicate that a visual stimulus which during training had been presented briefly before (group FORWARD) an aversive event, later on induces a potentiation of the startle responses (*t*_33_ = 2.91, *p* = 0.006) indicating negative implicit valence. In the FORWARD group, startle potentiation by CS+ and CS− did not differ (*t*_33_ = 0.72, *p* = 0.479). Importantly, however, if a visual stimulus had been following an aversive event during training (group BACKWARD), the startle response is attenuated in its presence (*t*_32_ = −2.1, *p* = 0.044), indicating implicit positive valence (i.e. safety). Supportively, the attenuation of startle by CS+ was stronger than by the CS− (*t*_32_ = −2.75, *p* = 0.010). Training with a long ISI (group CONTROL) did not subsequently affect the startle responses (*t*_33_ = −0.13, *p* = 0.894). Consistently, in the CONTROL group, startle responses in the presence of CS+ did not differ from the one in the presence of CS− (*t*_33_ = −0.71, *p* = 0.481).

**Figure 2.**
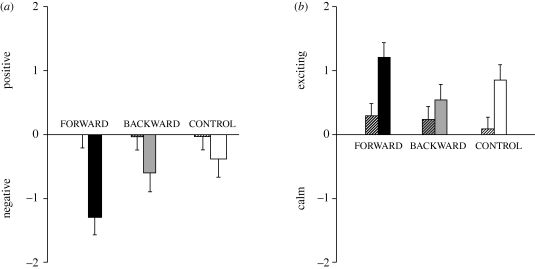
Bars represent mean scores (with standard errors) of (*a*) valence and (*b*) arousal ratings of the CS+ with the neutral value (i.e. 5) subtracted from the ratings. Hatched bars represent the ratings before the acquisition phase (i.e. conditioning) and filled bars after the acquisition phase. Black fill represents the FORWARD group, grey fill the BACKWARD group and white fill the CONTROL group. Negative values represent negative valence and low arousal, whereas positive values represent positive valence and high arousal. (*a*) Ratings of valence before training were neutral and after training were consistently negative indicating that the CS+ acquired negative explicit valence, independent of event-timing. (*b*) Ratings of arousal were in all three groups higher after compared with that before training, indicating that the CS+ became arousing independent of event-timing.

Exploiting the capacity of humans for explicit reports, we asked our participants to rate the valence and the arousal of the visual stimuli before and after the training phase. Importantly, both stimulus valence and arousal were rated as neutral before the training phase and the ratings at this time did not differ significantly between CS+, CS− and NEW. However, as indicated by significant interactions of stimulus × time (*F*_2,196_ = 22.43, *p* = 0, GG-ε = 0.91) and of stimulus × time × group (*F*_4,196_ = 4.12, *p* = 0.004, GG-ε = 0.91), CSs changed their valence through training (figure 2*a*). Comparisons within groups revealed that the CS+ was rated as ‘emotionally’ more negative after both forward *and* backward training compared with its initial pre-training rating (FORWARD: *t*_33_ = 5.55, *p* < 0.001; BACKWARD: *t*_32_ = 2.3, *p* = 0.028), but not after the control training (CONTROL: *t*_33_ = 1.23, *p* = 0.226). Comparisons between groups indicate that the FORWARD CS+ after training was rated as more negative when compared with the CONTROL CS+ (*t*_66_ = 2.34, *p* = 0.023), while this difference was not found before training (*t*_66_ = −0.11, *p* = 0.910). Although the valence of the BACKWARD CS+ after training valence did not differ significantly from the CONTROL CS+ valence after training (*t*_65_ = 0.54, *p* = 0.590), we note that both the FORWARD CS+ apparently acquired negative valence (FORWARD: *t*_33_ = −4.65, *p* = 0) and that the BACKWARD CS+ also acquired negative valence, which just failed to reach significance (BACKWARD: *t*_32_ = −1.95, *p* = 0.060); no such trend was seen for the CONTROL CS+ (*t*_33_ = −1.4, *p* = 0.172). This is confirmed by analyses of the differences between pre- and post-training scores on the basis of differences between the valence of the forward CS+ or the backward CS+ and the control CS+ revealed for both the FORWARD (post-training (forward − control) − pre-training (forward − control): *t*_33_ = 4.71, *p* < 0.001) and the BACKWARD group (post-training (backward − control) − pre-training (backward − control), *t*_32_ = 2.42, *p* = 0.021), arguing that the CS+ acquired negative explicit valence in both groups.

For arousal ratings, the ANOVA also indicated a significant stimulus × time interaction (*F*_2,196_ = 16.69, *p* = 0, GG-ε = 0.91), but the interaction stimulus × time × group was not significant (*F*_4,196_ = 0.56, *p* = 0.673, GG-ε = 0.91; figure 2*b*). Follow-up tests indicate that both the FORWARD CS+ and the CONTROL CS+ were rated as more arousing after the training phase compared with their initial pre-training ratings (FORWARD: *t*_33_ = − 3.14, *p* = 0.004; CONTROL: *t*_33_ = − 3.06, *p* = 0.004); this was not the case for the BACKWARD CS+ (*t*_32_ =−1.26, *p* = 0.216). There were no significant differences comparing FORWARD or BACKWARD CS+ arousal with CONTROL CS+ arousal (FORWARD: *t*_66_ = −1.13, *p* = 0.263; BACKWARD: *t*_65_ = 0.93, *p* = 0.355). However, in absolute terms, the FORWARD, the BACKWARD and the CONTROL CS+ acquired higher arousal since the arousal ratings after training did significantly differ from 0 (FORWARD: *t*_33_ = 4.91, *p* < 0.001; BACKWARD: *t*_32_ = 2.03, *p* = 0.051; CONTROL: *t*_33_ = 4.4, *p* < 0.001).

## 4. Discussion

Our findings reveal that event-timing determines the implicit valence of a CS in an opponent manner. On the one hand, a stimulus *signalling* an aversive event later potentiates startle responses, indicating that this stimulus acquired negative implicit valence. This result replicates animal as well as human fear conditioning studies (Lipp *et al*. 1994; Grillon 2002). On the other hand, a stimulus *following* an aversive event later attenuates startle responses, indicating that this stimulus acquired positive implicit valence. This new finding suggests that a stimulus associated with pain relief may activate reward circuits (Seymour *et al*. 2005; Leknes & Tracey 2008; Brischoux *et al*. 2009). Importantly, this observed opponency does not seem to be related to the omission of an expected aversive event (Dickinson & Dearing 1979), but rather to its termination (Solomon 1980; Seymour *et al*. 2005), since participants could correctly verbalize the association between the CS+ and the aversive US after both kinds of training. Finally, in humans this timing-dependent opponency appears restricted to implicit processes assessed on the basis of startle modulation. Explicit ratings of valence did not show timing-dependent opponency as both stimuli were rated as ‘emotionally negative’.

The observed timing-dependent bidirectional modulation of human startle behaviour conforms to the opponent-process theory of acquired motivation (Solomon 1980), which suggests that an aversive stimulus such as a painful electric shock generates two opponent processes: an initial negative affect upon onset and an after-process entailing the opposite state, i.e. positive affect. A stimulus presented after the aversive event thus may become associated with this latter process and therefore acquire positive valence. Please note however that the present experiment cannot directly verify these assumed opponent processes.

Notably, on the neural level our results concerning startle modulation could be explained on the basis of spike timing-dependent plasticity (Drew & Abbott 2006). That is, the temporal sequence of two inputs determines whether synapses are potentiated or depressed. This process can conceivably act at behaviourally relevant time scales (Abbott & Nelson 2000; Drew & Abbott 2006), in particular, if it was at operation in the amygdala and/or the dopamine neurons of NAcc (Brischoux *et al*. 2009), both structures relevant for associative startle response modulation (Davis 2006; Schneider & Spanagel 2008). Additionally, converging evidence indicates that potentiation or depression of synaptic firing in both amygdala (Delgado *et al*. 2006; Kim & Jung 2006) and NAcc (Wise 2004) are underlying mechanisms of associative memory. However, while amygdala responses have been found to habituate rapidly because of repeated stimulation (Quirk *et al*. 1997; LaBar *et al*. 1998; Büchel & Dolan 2000), no such results are reported for the NAcc. Given that we averaged across repeated extinction trials, this difference might explain why we observed no difference in startle amplitude in the presence of CS+ versus CS− after forward conditioning, but did find such a difference after backward conditioning. We further note that, in flies, effects of backward conditioning are apparently more stable over extended retention periods than the effects of forward conditioning (Yarali *et al*. 2008).

Importantly, our results suggest an event-timing-specific dissociation of implicit from explicit valence. Based on dual-process theories (e.g. Strack & Deutsch 2004), it might be speculated that after the offset of an aversive event, the impulsive, implicit system processes the experienced relief while the reflective, explicit system processes the overall aversiveness of the event. After all, the electric shock was painful and therefore aversive. This may be why a previously neutral stimulus presented briefly after an aversive event acquires positive implicit valence but nevertheless is explicitly evaluated as negative.

Further analyses of the implicit reward-like after-effects of aversive events in humans seem desirable in particular in the context of psychopathologies, e.g. anxiety disorders or drug addiction, including its modulation by genotype (see Yarali *et al*. 2009). For example, stimuli associated with the offset of a panic attack (e.g. the clinic or a physician) or the offset of withdrawal symptoms (e.g. the drug intake environment) may contribute to the maintenance of the disorder because they become appetitive and will be approached. Finally, although speculative, reward-like after-effects of aversive events may contribute to our understanding of ‘paradoxical’ human behaviours, like approaching stimuli that are explicitly evaluated as negative or dangerous (e.g. rollercoaster ride or bungee jumping).

In summary, the present study demonstrates that event-timing in humans crucially determines the implicit valence of a conditioned stimulus associated with an aversive event. Notably, stimuli associated with the offset of the aversive event acquire positive implicit valence. Moreover, the behaviour mediated by the impulsive, implicit system can dissociate from the expression of the reflective, explicit system. These findings should prompt studies to clarify the psychological and neuronal mechanisms behind the processes involved in event-timing and their implicit–explicit dissociation.
